# Differences between physicians' and nurse practitioners' viewpoints on reasons for clozapine underprescription

**DOI:** 10.1002/brb3.1318

**Published:** 2019-05-29

**Authors:** Cynthia Okhuijsen‐Pfeifer, Dan Cohen, Jan P. A. M. Bogers, Cato M. H. de Vos, Elianne A. H. Huijsman, René S. Kahn, Jurjen J. Luykx

**Affiliations:** ^1^ Department of psychiatry Brain Center Rudolf Magnus, University Medical Center Utrecht, Utrecht University Utrecht The Netherlands; ^2^ MHO GGZ‐NHN Heerhugowaard The Netherlands; ^3^ Dutch Clozapine Collaboration Group Castricum The Netherlands; ^4^ MHO Rivierduinen, High Care Clinics Oegstgeest The Netherlands; ^5^ Department of Psychiatry Icahn School of Medicine at Mount Sinai New York City New York; ^6^ Department of translational neuroscience University Medical Center Utrecht, Utrecht University Utrecht The Netherlands; ^7^ GGNet Mental Health Apeldoorn The Netherlands

**Keywords:** antipsychotic agents, clozapine, nurse practitioners, physician's practice patterns, schizophrenia spectrum and other psychotic disorders

## Abstract

**Introduction:**

Clozapine (CLZ) is the only proven effective therapy for treatment‐resistant schizophrenia, but it is underutilized across the globe. Previous findings suggest a lack of experience with CLZ prescription and concerns about CLZ's pharmacological characteristics are the prime reasons for CLZ underutilization. To our knowledge, it is currently unknown whether the reasons for underutilization and suggested solutions differ between physicians and nurse practitioners. Such differences are important as nurse practitioners are becoming increasingly involved in prescribing CLZ.

**Methods:**

To examine to what degree physicians and nurse practitioners differ with regard to their take on reasons for CLZ underutilization and suggested solutions, an online questionnaire was distributed to physicians and nurse practitioners. The primary outcome was to compare the *patient*‐related and *prescriber*‐related reasons for CLZ underprescription between physicians and nurse practitioners, while secondary outcome measures included the potential solutions to prevent this underprescription.

**Results:**

Physicians (*N* = 112) and nurse practitioners (*N* = 41) agreed that the two most common reasons for underprescription (*patient*‐related and *prescriber*‐related) were refusal to undergo regular blood tests and side‐effect concerns. They also agreed that the third most common *prescriber*‐related reason was medical complications. Physicians rated patients' unwillingness to switch medication as the third most common reason for CLZ underprescription, whereas nurse practitioners rated refusal to undergo baseline bloodtests as the third most common reason. The solutions to reduce underprescription largely corresponded between both groups.

**Conclusions:**

We conclude that slight differences exist between physicians' and nurse practitioners' viewpoints on patient‐related and prescriber‐related reasons for CLZ underprescription. Future research projects should involve patients to elucidate whether the patient‐related factors put forward by prescribers align with the patients' opinions.

## 1 INTRODUCTION

Clozapine (CLZ) is the only proven effective therapy for treatment‐resistant schizophrenia and even shows superior efficacy as a first‐ or second‐line treatment for this disorder (Okhuijsen‐Pfeifer et al., 2018), but the compound is underutilized across the globe (Howes et al., 2012). In a recent systematic review of reasons for CLZ underutilization (Verdoux, Quiles, Bachmann, & Siskind, 2018), the authors indicate a lack of experience with CLZ prescription and concerns about CLZ's pharmacological characteristics to be the prime reasons for CLZ underutilization. The implementation of CLZ clinics, simplification of blood monitoring, educational sessions about CLZ for prescribers and contact with experienced prescribers were suggested as potential solutions (Verdoux et al., 2018). The articles included in the review focussed on physicians' prescription attitudes. To our knowledge, it is currently unknown whether the reasons for underutilization and potential solutions differ between physicians and nurse practitioners. Disentangling such potential differences is important as nurse practitioners are becoming increasingly involved in prescribing CLZ across the globe. Nurse practitioners have the legal capacity to prescribe medications and work under the supervision of a medical doctor. We hypothesized their opinions about (underutilization of) CLZ and potential solutions are different from medical doctors' viewpoints, possibly because on average they spend more time with a specific patient than medical doctors. Therefore, we aimed to compare reasons and solutions for CLZ underutilization between physicians and nurse practitioners.

## 2 EXPERIMENTAL PROCEDURES

An online questionnaire was distributed to physicians and nurse practitioners through professional associations, academic hospitals, mental health centres, and other platforms in Flanders (the Dutch speaking part of Belgium) and the Netherlands. To our knowledge, this was the first time a questionnaire about CLZ prescription habits was circulated among prescribers in these areas. The questionnaire was based on previously used questionnaires (Gee, Vergunst, Howes, & Taylor, 2014; Nielsen, Dahm, Lublin, & Taylor, 2010). All relevant questions were used from these questionnaires, without modifying them. The current questionnaire was validated using forward and backward translations. Discrepancies were resolved by the authors during consensus meetings. For all analyses and data processing, SPSS version 25.0 (RRID:SCR_002865) was used. Differences between the two groups were tested using Fisher's exact test (*α* = 0.05). The primary outcome was to compare the patient‐related and prescriber‐related reasons for CLZ underprescription between physicians and nurse practitioners, while secondary outcome measures included the potential solutions to prevent this underprescription. To look into these research questions, percentages of physicians and nurse practitioners who rated a reason or solution as “frequent” or “very frequent” were calculated and compared in a top 3 per prescriber. The participants were allowed to rate all reasons and solutions from “not at all” to “very frequent” (5 categories). When a reason for delaying the initiation of CLZ was related to side effects, an open text box was available for participants to indicate what side effects they perceived as most involved in causing this delay. In addition, the ratios between patient‐related and prescriber‐related reasons that were rated as “frequent” or “very frequent” were calculated to investigate whether prescribers feel that the delay is mainly due to *patient*‐ or *prescriber*‐related reasons.

## 3 RESULTS

For all statistical tests mentioned in the results below, Fisher's exact test was used. One hundred and twelve physicians (88 psychiatrists and 24 psychiatrists in training) and 41 nurse practitioners (1 in training) completed the questionnaire. As this questionnaire was published online, the response rate is unknown. Experience (measured in years) with prescribing CLZ was similar between physicians and nurse practitioners: the mean (standard deviation) was *M = *13.5 (*SD = *9.5) years for physicians versus *M = *14.3 (*SD = *6.9) for nurse practitioners (*p* = 0.533). On a similar note, 85% of the physicians versus 90% of the nurse practitioners (*p* = 0.098) indicated to be at least fairly familiar with the current CLZ guidelines. In sum, the results below cannot be explained by baseline differences.

In the online questionnaire, physicians reported a mean (standard deviation; percentage) caseload of *M = *97.4 (*SD = *227.3) schizophrenia spectrum disorder patients, of whom *M = *2.7 (*SD = *5.0; 3%) in their opinion should use CLZ, but currently did not; for nurse practitioners the mean caseload of schizophrenia spectrum disorder patients was *M = *76.2 (*SD = *188.8) of whom *M = *7.9 (*SD = *11.05; 10%) should use CLZ. This estimated number of subjects who should be using CLZ but were not, was significantly lower in physicians' caseloads than in nurse practitioners' caseloads (*p = *0.023). Interestingly, CLZ was considered the third antipsychotic of choice for all indications by both groups, but over 50% of the prescribers (47% of physicians and 71% of nurse practitioners) have patients in their caseloads who have used more than three antipsychotics.

Physicians and nurse practitioners agreed that the two most common reasons for underprescription (patient‐related and prescriber‐related) were refusal to undergo regular blood tests and side‐effect concerns. They agreed that the third most common *prescriber*‐related reason was possible medical complications. They disagreed on the third most common *patient*‐related reason: physicians rated patients' unwillingness to switch medication as the third most common reason for CLZ underprescription, whereas nurse practitioners rated refusal to undergo baseline blood tests as the third most common reason.

Side effects concerns is a broad term used for concerns about all potential CLZ‐emergent adverse reactions. Both physicians and nurse practitioners worried most about the following three categories of side effects (ranked by decreasing frequencies): weight gain and metabolic symptoms; agranulocytosis and related disorders; and cardiovascular adverse reactions. Interestingly, hypersalivation did not make the top 3.

The ratio between the number of patient‐related reasons and prescriber‐related reasons for underprescription was 2 for physicians versus 1.3 for nurse practitioners, suggesting that physicians attribute the underprescription more to the patients, while nurse practitioners attribute the underprescription fairly equally to patients and prescribers.

Potential solutions (very) frequently brought forward by physicians were “personnel to guide CLZ initiation in outpatients” (by 55% of physicians), “sufficient time to guide CLZ initiation in outpatients” (51%), and “sufficient beds to guide CLZ in inpatients” (25%). Potential solutions brought forward by nurse practitioners were “sufficient time to guide CLZ initiation in outpatients” (by 49% of nurse practitioners), “personnel to guide CLZ initiation in outpatients” (44%), and “extra personnel for baseline blood tests” (17%).

## 4 CONCLUSIONS

We conclude that slight differences exist between physicians' and nurse practitioners' viewpoints on patient‐related and prescriber‐related reasons for CLZ underprescription. Physicians are more concerned that patients do not want to switch medication, while nurse practitioners think that patients will refuse to undergo baseline blood tests. Nurse practitioners attribute underprescription equally to both patients and prescribers while physicians attribute underprescription more to patients. The solutions to reduce underprescription largely corresponded between both groups. The main difference was that physicians mentioned admissions could help boost prescription rates while nurse practitioners believed that extra personnel to obtain blood tests would be helpful. In contrast to the systematic review mentioned in the introduction (Verdoux et al., 2018), this study highlights concerns with the pharmacological characteristics of CLZ, but not the lack of personal prescribing experience, as an important reason for CLZ underprescription.

Our findings have implications for future research and clinical practice. Upcoming research projects should involve patients to elucidate whether the patient‐related factors put forward by prescribers align with patients' opinions. Most importantly, when implementing strategies in clinical practice to reduce CLZ underprescription, the highlighted differences between physicians and nurse practitioners should be included to increase success likelihoods of such strategies. For example, based on the current findings extra personnel to obtain blood tests may be a more optimal solution to boost prescription by nurse practitioners, whereas for physicians sufficient beds to guide CLZ initiation may be a more helpful strategy.

## CONFLICT OF INTEREST

DC and JB are board members of the nonprofit Dutch Clozapine Collaboration Group. JB received an unrestricted grant from Pfizer Biopharmaceutical Company. RK declares personal fees for consultancy from Alkermes, Minerva Neuroscience, Gedeon Richter, and Otsuka; and personal (speaker) fees from Otsuka/Lundbeck. All other authors declare no conflict of interest.

## Data Availability

Data available on request from the authors.
